# Evaluation of 3D-Jury on CASP7 models

**DOI:** 10.1186/1471-2105-8-304

**Published:** 2007-08-21

**Authors:** László Kaján, Leszek Rychlewski

**Affiliations:** 1 BioInfoBank Institute, ul. Limanowskiego 24 A, 60-744 Poznań, Poland; 2 Bioinformatics Unit, Department of Physics, Adam Mickiewicz University, ul. Umultowska 85, 61-614 Poznań, Poland

## Abstract

**Background:**

3D-Jury, the structure prediction consensus method publicly available in the Meta Server , was evaluated using models gathered in the 7^*th *^round of the Critical Assessment of Techniques for Protein Structure Prediction (CASP7). 3D-Jury is an automated expert process that generates protein structure meta-predictions from sets of models obtained from partner servers.

**Results:**

The performance of 3D-Jury was analysed for three aspects. First, we examined the correlation between the 3D-Jury score and a model quality measure: the number of correctly predicted residues. The 3D-Jury score was shown to correlate significantly with the number of correctly predicted residues, the correlation is good enough to be used for prediction. 3D-Jury was also found to improve upon the competing servers' choice of the best structure model in most cases. The value of the 3D-Jury score as a generic reliability measure was also examined. We found that the 3D-Jury score separates bad models from good models better than the reliability score of the original server in 27 cases and falls short of it in only 5 cases out of a total of 38. We report the release of a new Meta Server feature: instant 3D-Jury scoring of uploaded user models.

**Conclusion:**

The 3D-Jury score continues to be a good indicator of structural model quality. It also provides a generic reliability score, especially important for models that were not assigned such by the original server. Individual structure modellers can also benefit from the 3D-Jury scoring system by testing their models in the new instant scoring feature  available in the Meta Server.

## Background

The number of protein structure prediction servers has increased over the past years [1]. The use of many different methods to predict the structure of a protein is now state-of-the-art in protein structure prediction [2]. However, the number of available servers, taken together with the number of models returned exceeds the limit a human researcher is likely to scan. Fortunately, structure prediction meta-servers address this problem: they gather models from various other servers and employ automated processes successfully applied by human experts in order to deliver a correct prediction [1]. Since existing structure prediction servers are constantly upgraded while new servers appear, it is necessary to re-evaluate the fitness of the aforementioned expert processes.

The latest, 7^*th *^round of the Critical Assessment of Techniques for Protein Structure Prediction [3] has provided us with a fair amount of structure prediction server models. With the help of the Structure Prediction Meta Server [4], we have evaluated the servers returning these models using the same protocols as in previous Livebench experiments [5], results are available at [6].

Standard evaluation methods take into account the first (top ranked) model of the prediction servers. The Meta Server assigns a new reliability score to each model using 3D-Jury [7]. This score can be used to re-rank the models and thus affect the evaluation results. The aim of the present work was to verify the continued applicability of this model ranking method, focusing on the version available on-line. We were interested in answering the following three questions: Can we use 3D-Jury to estimate model quality? Does 3D-Jury select a model more accurate than the choice of the generating server? Could the 3D-Jury score be used as a generic model reliability score?

## Results and Discussion

### 3D-Jury score correlates with the number of correctly predicted residues

The correlation of the 3D-Jury score (*Jscore*) with model quality is of fundamental importance to the operation of the Meta Server. Therefore we first examined the correlation of the 3D-Jury score returned by the default on-line version of 3D-Jury: 3J_1,*A *_(see Methods: 3D-Jury operating modes), with the number of correctly predicted residues (NCα≤3.5Å
 MathType@MTEF@5@5@+=feaafiart1ev1aaatCvAUfKttLearuWrP9MDH5MBPbIqV92AaeXatLxBI9gBaebbnrfifHhDYfgasaacH8akY=wiFfYdH8Gipec8Eeeu0xXdbba9frFj0=OqFfea0dXdd9vqai=hGuQ8kuc9pgc9s8qqaq=dirpe0xb9q8qiLsFr0=vr0=vr0dc8meaabaqaciaacaGaaeqabaqabeGadaaakeaacqWGobGtdaWgaaWcbaGaem4qam0aaSbaaWqaaGGaciab=f7aHbqabaWccqGHKjYOcqaIZaWmcqGGUaGlcqaI1aqntCvAUfeBSjuyZL2yd9gzLbvyNv2CaeHbhv2BYDwAHbaceiGaa4xXaaqabaaaaa@413E@).

**Figure 1:**
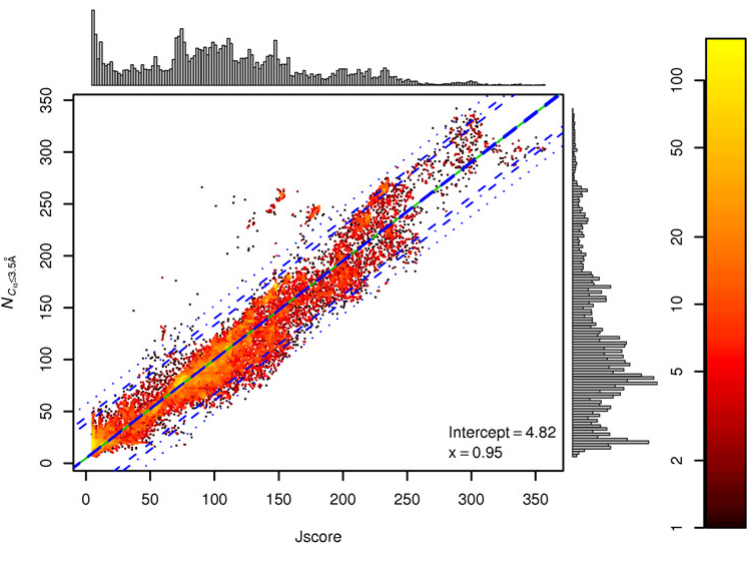
**Correlation of 3D-Jury score with the number of correctly predicted C_*α *_atoms**. NCα≤3.5Å
 MathType@MTEF@5@5@+=feaafiart1ev1aaatCvAUfKttLearuWrP9MDH5MBPbIqV92AaeXatLxBI9gBaebbnrfifHhDYfgasaacH8akY=wiFfYdH8Gipec8Eeeu0xXdbba9frFj0=OqFfea0dXdd9vqai=hGuQ8kuc9pgc9s8qqaq=dirpe0xb9q8qiLsFr0=vr0=vr0dc8meaabaqaciaacaGaaeqabaqabeGadaaakeaacqWGobGtdaWgaaWcbaGaem4qam0aaSbaaWqaaGGaciab=f7aHbqabaWccqGHKjYOcqaIZaWmcqGGUaGlcqaI1aqntCvAUfeBSjuyZL2yd9gzLbvyNv2CaeHbhv2BYDwAHbaceiGaa4xXaaqabaaaaa@413E@ – the number of C_*α *_atoms predicted within 3.5 Å from their respective locations in the crystal structure; *Jscore *– 3J_1,*A *_score; solid green line – prediction of linear model LM_1_; blue longdash lines: confidence interval at 95% confidence level; blue dashed lines: prediction interval at 90% confidence level; blue dotdash lines: prediction interval at 95% confidence level; blue dotted lines: prediction interval at 99% confidence level; x – slope; the colour bar is key to the approximate density of models A linear model (LM_1_) was fitted to the 3D-Jury score vs. NCα≤3.5Å
 MathType@MTEF@5@5@+=feaafiart1ev1aaatCvAUfKttLearuWrP9MDH5MBPbIqV92AaeXatLxBI9gBaebbnrfifHhDYfgasaacH8akY=wiFfYdH8Gipec8Eeeu0xXdbba9frFj0=OqFfea0dXdd9vqai=hGuQ8kuc9pgc9s8qqaq=dirpe0xb9q8qiLsFr0=vr0=vr0dc8meaabaqaciaacaGaaeqabaqabeGadaaakeaacqWGobGtdaWgaaWcbaGaem4qam0aaSbaaWqaaGGaciab=f7aHbqabaWccqGHKjYOcqaIZaWmcqGGUaGlcqaI1aqntCvAUfeBSjuyZL2yd9gzLbvyNv2CaeHbhv2BYDwAHbaceiGaa4xXaaqabaaaaa@413E@ of 19,558 models. The residual standard error is 20.15. The 95% confidence interval as well as prediction intervals for 90%, 95% and 99% confidence levels are indicated on the figure. The vertical and horizontal histograms show the distributions of NCα≤3.5Å
 MathType@MTEF@5@5@+=feaafiart1ev1aaatCvAUfKttLearuWrP9MDH5MBPbIqV92AaeXatLxBI9gBaebbnrfifHhDYfgasaacH8akY=wiFfYdH8Gipec8Eeeu0xXdbba9frFj0=OqFfea0dXdd9vqai=hGuQ8kuc9pgc9s8qqaq=dirpe0xb9q8qiLsFr0=vr0=vr0dc8meaabaqaciaacaGaaeqabaqabeGadaaakeaacqWGobGtdaWgaaWcbaGaem4qam0aaSbaaWqaaGGaciab=f7aHbqabaWccqGHKjYOcqaIZaWmcqGGUaGlcqaI1aqntCvAUfeBSjuyZL2yd9gzLbvyNv2CaeHbhv2BYDwAHbaceiGaa4xXaaqabaaaaa@413E@ and 3D-Jury scores respectively.

3D-Jury scores correlate with the number of correctly predicted residues (NCα≤3.5Å
 MathType@MTEF@5@5@+=feaafiart1ev1aaatCvAUfKttLearuWrP9MDH5MBPbIqV92AaeXatLxBI9gBaebbnrfifHhDYfgasaacH8akY=wiFfYdH8Gipec8Eeeu0xXdbba9frFj0=OqFfea0dXdd9vqai=hGuQ8kuc9pgc9s8qqaq=dirpe0xb9q8qiLsFr0=vr0=vr0dc8meaabaqaciaacaGaaeqabaqabeGadaaakeaacqWGobGtdaWgaaWcbaGaem4qam0aaSbaaWqaaGGaciab=f7aHbqabaWccqGHKjYOcqaIZaWmcqGGUaGlcqaI1aqntCvAUfeBSjuyZL2yd9gzLbvyNv2CaeHbhv2BYDwAHbaceiGaa4xXaaqabaaaaa@413E@): the correlation coefficient is 0.95. A linear model (LM_1_) is presented on Figure 1. The residual error, 20.15, is low enough to enable meaningful estimation of the number of correctly positioned residues.

**Figure 2:**
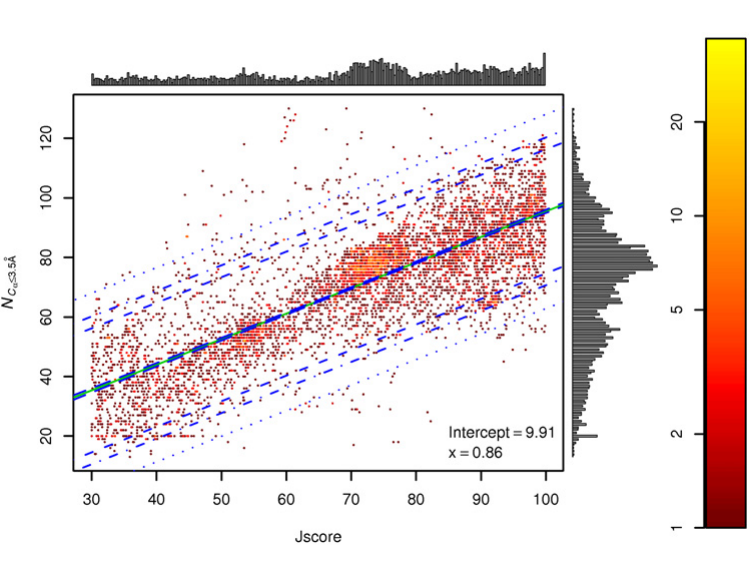
**Correlation of 3D-Jury score in the [30–100) range with the number of correctly predicted C_*α *_atoms**. NCα≤3.5Å
 MathType@MTEF@5@5@+=feaafiart1ev1aaatCvAUfKttLearuWrP9MDH5MBPbIqV92AaeXatLxBI9gBaebbnrfifHhDYfgasaacH8akY=wiFfYdH8Gipec8Eeeu0xXdbba9frFj0=OqFfea0dXdd9vqai=hGuQ8kuc9pgc9s8qqaq=dirpe0xb9q8qiLsFr0=vr0=vr0dc8meaabaqaciaacaGaaeqabaqabeGadaaakeaacqWGobGtdaWgaaWcbaGaem4qam0aaSbaaWqaaGGaciab=f7aHbqabaWccqGHKjYOcqaIZaWmcqGGUaGlcqaI1aqntCvAUfeBSjuyZL2yd9gzLbvyNv2CaeHbhv2BYDwAHbaceiGaa4xXaaqabaaaaa@413E@ – the number of C_*α *_atoms predicted within 3.5 Å from their respective locations in the crystal structure; *Jscore *– 3J_1,*A *_score; solid green line – prediction of linear model LM_2_; blue longdash lines: confidence interval at 95% confidence level; blue dashed lines: prediction interval at 90% confidence level; blue dotdash lines: prediction interval at 95% confidence level; blue dotted lines: prediction interval at 99% confidence level; x – slope; the colour bar is key to the approximate density of models A linear model (LM_2_) was fitted to the 3D-Jury score vs. NCα≤3.5Å
 MathType@MTEF@5@5@+=feaafiart1ev1aaatCvAUfKttLearuWrP9MDH5MBPbIqV92AaeXatLxBI9gBaebbnrfifHhDYfgasaacH8akY=wiFfYdH8Gipec8Eeeu0xXdbba9frFj0=OqFfea0dXdd9vqai=hGuQ8kuc9pgc9s8qqaq=dirpe0xb9q8qiLsFr0=vr0=vr0dc8meaabaqaciaacaGaaeqabaqabeGadaaakeaacqWGobGtdaWgaaWcbaGaem4qam0aaSbaaWqaaGGaciab=f7aHbqabaWccqGHKjYOcqaIZaWmcqGGUaGlcqaI1aqntCvAUfeBSjuyZL2yd9gzLbvyNv2CaeHbhv2BYDwAHbaceiGaa4xXaaqabaaaaa@413E@ of 6,710 models. The residual standard error is 13.37. The 95% confidence interval as well as prediction intervals for 90%, 95% and 99% confidence levels are indicated on the figure. The vertical and horizontal histograms show the distributions of NCα≤3.5Å
 MathType@MTEF@5@5@+=feaafiart1ev1aaatCvAUfKttLearuWrP9MDH5MBPbIqV92AaeXatLxBI9gBaebbnrfifHhDYfgasaacH8akY=wiFfYdH8Gipec8Eeeu0xXdbba9frFj0=OqFfea0dXdd9vqai=hGuQ8kuc9pgc9s8qqaq=dirpe0xb9q8qiLsFr0=vr0=vr0dc8meaabaqaciaacaGaaeqabaqabeGadaaakeaacqWGobGtdaWgaaWcbaGaem4qam0aaSbaaWqaaGGaciab=f7aHbqabaWccqGHKjYOcqaIZaWmcqGGUaGlcqaI1aqntCvAUfeBSjuyZL2yd9gzLbvyNv2CaeHbhv2BYDwAHbaceiGaa4xXaaqabaaaaa@413E@ and 3D-Jury scores respectively. The 30 to 100 3D-Jury score range was chosen to represent difficult targets.

A better model (LM_2_) can be obtained by fitting to the [30, 100) 3D-Jury score range only. This range represents difficult targets. Figure 2 shows the linear model obtained. The residual error is 13.37, offering narrower, better prediction intervals for the number of correctly positioned residues.

As an example to the use of LM_2_, let's assume that our model has 3D-Jury score 44.5. We can expect to have 13 to 82 well positioned residues in this model on the 99% confidence level, 21 to 74 on the 95% confidence level. For a score of 59 the 99% prediction interval for the number of correct residues is 26–94, the 95% prediction interval is narrower: 34–86.

A key to which residues are likely to be well-positioned is provided on the model-centred 3D-Jury page, accessible by selecting a model in the Model column of the main 3D-Jury page. Here, residues that are likely to be correctly positioned would have grey background at the corresponding positions of most of the *other *aligned models, forming a column of grey background.

### 3D-Jury improves overall server prediction results

We examined whether 3D-Jury could improve overall server performance by selecting a better model when multiple models are returned by a prediction server. We tested four operating modes of 3D-Jury: 3J_1,*A *_– uses *one *model of the *default *servers (a mode typical for on-line predictions); 3J_*a*,*A *_– *all *models of *default *servers; 3J_1,*C *_– *one *model of *all *servers; 3J_*a,C *_– *all *models of *all *servers. We have computed the MaxSub score (*MaxS*) [8] of 25,215 models for this analysis. Four 3D-Jury scores (*Jscore*) were also computed for each model, respective to the four 3D-Jury operating modes mentioned above. The servers' choice of the best model was evaluated by summing the *MaxS*' of the *first *models returned for each target. The four 3D-Jury variants' choice of the best model was evaluated by summing the *MaxS*' of the models with the highest respective 3D-Jury score for each target. We also summed up the highest *MaxS *score for each target, giving an upper limit to possible improvements. Results for 3J_1,*A *_are presented in Table 1, column *Q*_%_. The order of the five model ranking approaches is revealed by the grand total of *MaxS*: 3J_*a*,*C *_(20,006) > 3J_1,*C *_(19,983) > 3J_1,*A *_(19,690) > 3_*a,A *_(19,655) > first server model (19,039) (the sum of *MaxS *over the highest scoring models is 20,718). Table 1, column *N*_*j *_shows the number of targets where 3J_1,*A *_made a better choice about the best model than the original server. In the case of pmodeller6 [9] and 3dpro [10], we can see that 3D-Jury 3J_1,*A *_predicts more targets better, but its overall performance is slightly worse than the original servers'. The reason for this is that 3J_1,*A*_'s more numerous choices of better models were not good enough to counteract its loss of MaxSub scores on the bad choices. In the case of inub [11] and BasD [12] the situation is inverse: 3J_1,*A *_improved fewer targets, but the net improvement is positive. For many servers the improvement – or worsening – of the targets is marginal (e.g. phyre-2 = 0.6%). Nevertheless we can see that even in these cases there is room for a 4 – 5% improvement (Table 1, column *Q*_%_, values in parentheses). Moreover, it appears that for at least 14 targets every server fails to pick the best model.

**Table 1:** Server prediction results improved by 3D-Jury. 3J_1,*A *_– the default on-line version of 3D-Jury, uses *one *model of the *default *servers [7]; *N*_*s *_– number of targets better predicted (in terms of MaxSub score) by the server; *N*_*j *_– number of targets better predictedby 3J_1,*A*_, in parentheses: number of improvable targets, i.e. those with a suboptimal choice of the first model; *Q*_%_, Q%max
 MathType@MTEF@5@5@+=feaafiart1ev1aaatCvAUfKttLearuWrP9MDH5MBPbIqV92AaeXatLxBI9gBaebbnrfifHhDYfgasaacH8akY=wiFfYdH8Gipec8Eeeu0xXdbba9frFj0=OqFfea0dXdd9vqai=hGuQ8kuc9pgc9s8qqaq=dirpe0xb9q8qiLsFr0=vr0=vr0dc8meaabaqaciaacaGaaeqabaqabeGadaaakeaacqWGrbqudaqhaaWcbaGaeiyjaucabaGaemyBa0MaemyyaeMaemiEaGhaaaaa@32FD@ – see Methods: Measures for comparing model selection methods Servers are ordered by N_*j*_-N_*s *_descending, three servers with ∑*MaxS*_*s *_= 0 are not shown. Servers not improved by the re-ranking of models (N_*s *_> N_*j*_) are shown in italics. 3J_1,*A *_selects better models on the whole for 50 servers out of the 56 shown, considering either *Q*_% _or the number of targets. Re-ranking of models by 3D-Jury does not improve the performance of 6 servers.

Server	*N*_*s*_	*N*_*j*_	Q%(Q%max) MathType@MTEF@5@5@+=feaafiart1ev1aaatCvAUfKttLearuWrP9MDH5MBPbIqV92AaeXatLxBI9gBaebbnrfifHhDYfgasaacH8akY=wiFfYdH8Gipec8Eeeu0xXdbba9frFj0=OqFfea0dXdd9vqai=hGuQ8kuc9pgc9s8qqaq=dirpe0xb9q8qiLsFr0=vr0=vr0dc8meaabaqaciaacaGaaeqabaqabeGadaaakeaacqWGrbqudaWgaaWcbaGaeiyjaucabeaakiabcIcaOiabdgfarnaaDaaaleaacqGGLaqjaeaacqWGTbqBcqWGHbqycqWG4baEaaGccqGGPaqkaaa@36EC@	*R*_*s*_	Server	*N*_*s*_	*N*_*j*_	Q%(Q%max) MathType@MTEF@5@5@+=feaafiart1ev1aaatCvAUfKttLearuWrP9MDH5MBPbIqV92AaeXatLxBI9gBaebbnrfifHhDYfgasaacH8akY=wiFfYdH8Gipec8Eeeu0xXdbba9frFj0=OqFfea0dXdd9vqai=hGuQ8kuc9pgc9s8qqaq=dirpe0xb9q8qiLsFr0=vr0=vr0dc8meaabaqaciaacaGaaeqabaqabeGadaaakeaacqWGrbqudaWgaaWcbaGaeiyjaucabeaakiabcIcaOiabdgfarnaaDaaaleaacqGGLaqjaeaacqWGTbqBcqWGHbqycqWG4baEaaGccqGGPaqkaaa@36EC@	*R*_*s*_
protinfo-ab [22]	9	32(54)	1.8(4.4)	24	raptor-ace [23]	18	27(50)	2.1(7.5)	8
3d-jigsaw [24]	9	31(38)	10.7(12.8)	51	fugue [25]	9	17(25)	5.4(9.0)	35
ma-opus-server	11	28(43)	9.0(13.2)	28	function [26]	14	21(40)	1.3(5.6)	11
sam_t06_server [27]	13	30(47)	7.7(13.4)	25	gtg [28]	4	11(16)	6.5(8.2)	54
caspita-fox [29]	11	27(37)	10.4(15.6)	40	huber-torda-server [30]	9	16(25)	8.3(10.6)	47
forecast-s	6	22(28)	5.9(8.6)	49	sp4 [31]	12	19(36)	3.2(7.6)	10
3d-jigsaw_populus [24]	13	28(38)	4.7(7.1)	48	sparks2 [31]	13	20(32)	3.7(7.5)	16
loopp [32]	14	29(39)	6.8(9.9)	33	ffas03 [13]	20	26(42)	1.5(9.2)	19
ma-opus-server2	5	20(32)	9.4(13.9)	52	frankenstein [33]	6	12(14)	4.9(7.1)	55
sam-t02 [34]	11	26(34)	4.1(6.8)	27	keasar-server [35]	19	25(52)	5.9(12.5)	34
3d-jigsaw_recom [24]	11	25(36)	4.1(7.1)	50	pmodeller6 [2]	30	36(59)	-1.2(9.0)	2
forte2 [36]	9	23(29)	7.2(11.2)	41	uni-eid_sfst	11	17(35)	3.4(8.3)	31
phyre-2 [19]	9	23(39)	0.6(3.8)	30	circle [26]	21	26(53)	0.1(5.9)	4
robetta [37]	20	34(63)	1.3(12.4)	6	foldpro [10]	10	15(30)	0.8(3.7)	15
genesilicometaserver [33]	16	29(44)	1.3(5.7)	13	fugmod [25]	12	16(22)	4.7(8.6)	37
mgenthreader [38]	11	24(39)	1.3(8.6)	26	sp3 [31]	15	18(30)	1.9(5.8)	9
karypis.srv [39]	12	24(38)	5.1(11.3)	43	3dpro [10]	11	13(32)	-0.5(4.2)	20
karypis.srv.2 [39]	12	24(42)	2.7(9.4)	46	ORFeus-2 [17]	17	19(44)	1.3(9.5)	29
sam-t99 [40]	14	26(39)	3.0(6.4)	42	raptor [23]	20	22(44)	2.5(10.6)	12
forte1 [36]	11	22(27)	7.5(12.0)	38	uni-eid bnmx	16	18(39)	0.5(7.2)	21
pdbblast [18]	10	21(33)	3.2(7.3)	39	metatasser [41]	16	17(40)	1.5(5.7)	14
rokky [42]	9	20(44)	5.7(11.6)	36	protinfo [22]	25	26(52)	1.7(9.9)	22
abipro	0	10(28)	79.5(213.4)	56	*distill [43]*	12	11(33)	-0.6(7.3)	53
fams [26]	21	31(50)	2.8(7.7)	7	*inub [44]*	16	15(38)	1.2(8.1)	17
nfold [16]	11	21(32)	4.8(9.7)	32	*pcons6 [2]*	26	22(50)	-2.3(6.0)	3
raptoress [23]	15	25(45)	5.4(5.4)	18	*famsd [26]*	23	18(36)	-1.1(4.4)	5
bilab-enable	13	22(36)	7.2(7.2)	44	*zhang-server [45]*	23	18(54)	-0.4(5.1)	1
3D-PSSM [19]	7	16(38)	5.2(12.4)	45	*BasD [12]*	23	16(42)	1.1(7.5)	23

### 3D-Jury scores as generic model reliability scores

In order to assess the advantage of using 3D-Jury scores as generic reliability scores we conducted a receiver operating characteristic (ROC) analysis adapted for CASP and Livebench [5] evaluation. The analysis shows how well a reliability score separates good models from bad ones, in terms of the average number of good models seen before encountering 1 to 11 bad models (tp¯
 MathType@MTEF@5@5@+=feaafiart1ev1aaatCvAUfKttLearuWrP9MDH5MBPbIqV92AaeXatLxBI9gBaebbnrfifHhDYfgasaacH8akY=wiFfYdH8Gipec8Eeeu0xXdbba9frFj0=OqFfea0dXdd9vqai=hGuQ8kuc9pgc9s8qqaq=dirpe0xb9q8qiLsFr0=vr0=vr0dc8meaabaqaciaacaGaaeqabaqabeGadaaakeaadaqdaaqaaiabdsha0jabdchaWbaaaaa@2F97@). We compared the 3D-Jury scores returned by the on-line version 3J_1,*A *_to the reliability scores of the original servers, when available. Results are shown in Table 2. The 3D-Jury score exceeds the original server score (tp¯R
 MathType@MTEF@5@5@+=feaafiart1ev1aaatCvAUfKttLearuWrP9MDH5MBPbIqV92AaeXatLxBI9gBaebbnrfifHhDYfgasaacH8akY=wiFfYdH8Gipec8Eeeu0xXdbba9frFj0=OqFfea0dXdd9vqai=hGuQ8kuc9pgc9s8qqaq=dirpe0xb9q8qiLsFr0=vr0=vr0dc8meaabaqaciaacaGaaeqabaqabeGadaaakeaadaqdaaqaaiabdsha0jabdchaWbaadaWgaaWcbaGaemOuaifabeaaaaa@30F0@) in 27 cases and falls short of it in only 5 cases out of the 38 analysed. The exceptions are pmodeller6 [9], pcons6 [2], ffas03 [13], inub [11] and shub [11].

**Table 2:** 3D-Jury receiver operating characteristic (ROC) analysis. tp¯R
 MathType@MTEF@5@5@+=feaafiart1ev1aaatCvAUfKttLearuWrP9MDH5MBPbIqV92AaeXatLxBI9gBaebbnrfifHhDYfgasaacH8akY=wiFfYdH8Gipec8Eeeu0xXdbba9frFj0=OqFfea0dXdd9vqai=hGuQ8kuc9pgc9s8qqaq=dirpe0xb9q8qiLsFr0=vr0=vr0dc8meaabaqaciaacaGaaeqabaqabeGadaaakeaadaqdaaqaaiabdsha0jabdchaWbaadaWgaaWcbaGaemOuaifabeaaaaa@30F0@ – average number of true positive (*tp*) models in the [0 – 10] false positive (*fp*) range, using the reliability score provided by the server as the discrimination threshold; tp¯J
 MathType@MTEF@5@5@+=feaafiart1ev1aaatCvAUfKttLearuWrP9MDH5MBPbIqV92AaeXatLxBI9gBaebbnrfifHhDYfgasaacH8akY=wiFfYdH8Gipec8Eeeu0xXdbba9frFj0=OqFfea0dXdd9vqai=hGuQ8kuc9pgc9s8qqaq=dirpe0xb9q8qiLsFr0=vr0=vr0dc8meaabaqaciaacaGaaeqabaqabeGadaaakeaadaqdaaqaaiabdsha0jabdchaWbaadaWgaaWcbaGaemOsaOeabeaaaaa@30E0@ – average number of *tp *in the [0 – 10] *fp *range using 3D-Jury score as the discrimination threshold; *J*_0 _– lowest 3D-Jury score before observing the first bad model; tpJ0
 MathType@MTEF@5@5@+=feaafiart1ev1aaatCvAUfKttLearuWrP9MDH5MBPbIqV92AaeXatLxBI9gBaebbnrfifHhDYfgasaacH8akY=wiFfYdH8Gipec8Eeeu0xXdbba9frFj0=OqFfea0dXdd9vqai=hGuQ8kuc9pgc9s8qqaq=dirpe0xb9q8qiLsFr0=vr0=vr0dc8meaabaqaciaacaGaaeqabaqabeGadaaakeaacqWG0baDcqWGWbaCdaWgaaWcbaGaemOsaO0aaSbaaWqaaiabicdaWaqabaaaleqaaaaa@31F5@ – number of good models at or above *J*_0 _score; *N*_*t *_– number of targets The table shows results for the on-line default version of 3D-Jury: 3J_1,*A*_. Servers are ordered by tp¯J
 MathType@MTEF@5@5@+=feaafiart1ev1aaatCvAUfKttLearuWrP9MDH5MBPbIqV92AaeXatLxBI9gBaebbnrfifHhDYfgasaacH8akY=wiFfYdH8Gipec8Eeeu0xXdbba9frFj0=OqFfea0dXdd9vqai=hGuQ8kuc9pgc9s8qqaq=dirpe0xb9q8qiLsFr0=vr0=vr0dc8meaabaqaciaacaGaaeqabaqabeGadaaakeaadaqdaaqaaiabdsha0jabdchaWbaadaWgaaWcbaGaemOsaOeabeaaaaa@30E0@ descending. Missing tp¯R
 MathType@MTEF@5@5@+=feaafiart1ev1aaatCvAUfKttLearuWrP9MDH5MBPbIqV92AaeXatLxBI9gBaebbnrfifHhDYfgasaacH8akY=wiFfYdH8Gipec8Eeeu0xXdbba9frFj0=OqFfea0dXdd9vqai=hGuQ8kuc9pgc9s8qqaq=dirpe0xb9q8qiLsFr0=vr0=vr0dc8meaabaqaciaacaGaaeqabaqabeGadaaakeaadaqdaaqaaiabdsha0jabdchaWbaadaWgaaWcbaGaemOuaifabeaaaaa@30F0@ values indicate servers that did not return reliability scores. Five servers with tp¯R>tp¯J
 MathType@MTEF@5@5@+=feaafiart1ev1aaatCvAUfKttLearuWrP9MDH5MBPbIqV92AaeXatLxBI9gBaebbnrfifHhDYfgasaacH8akY=wiFfYdH8Gipec8Eeeu0xXdbba9frFj0=OqFfea0dXdd9vqai=hGuQ8kuc9pgc9s8qqaq=dirpe0xb9q8qiLsFr0=vr0=vr0dc8meaabaqaciaacaGaaeqabaqabeGadaaakeaadaqdaaqaaiabdsha0jabdchaWbaadaWgaaWcbaGaemOuaifabeaakiabg6da+maanaaabaGaemiDaqNaemiCaahaamaaBaaaleaacqWGkbGsaeqaaaaa@3636@ are shown in italics. In order to assess 3D-Jury scores (*Jscore*) as reliability scores, we performed a ROC analysis adapted for CASP and Livebench data, comparing *Jscore *to the reliability scores provided by the servers. In terms of the average number of true positive models (tp¯
 MathType@MTEF@5@5@+=feaafiart1ev1aaatCvAUfKttLearuWrP9MDH5MBPbIqV92AaeXatLxBI9gBaebbnrfifHhDYfgasaacH8akY=wiFfYdH8Gipec8Eeeu0xXdbba9frFj0=OqFfea0dXdd9vqai=hGuQ8kuc9pgc9s8qqaq=dirpe0xb9q8qiLsFr0=vr0=vr0dc8meaabaqaciaacaGaaeqabaqabeGadaaakeaadaqdaaqaaiabdsha0jabdchaWbaaaaa@2F97@), the 3D-Jury score exceeds the original server score in 27 cases, it falls short of it in 5 cases out of the 38 analysed.

Server	tp¯R MathType@MTEF@5@5@+=feaafiart1ev1aaatCvAUfKttLearuWrP9MDH5MBPbIqV92AaeXatLxBI9gBaebbnrfifHhDYfgasaacH8akY=wiFfYdH8Gipec8Eeeu0xXdbba9frFj0=OqFfea0dXdd9vqai=hGuQ8kuc9pgc9s8qqaq=dirpe0xb9q8qiLsFr0=vr0=vr0dc8meaabaqaciaacaGaaeqabaqabeGadaaakeaadaqdaaqaaiabdsha0jabdchaWbaadaWgaaWcbaGaemOuaifabeaaaaa@30F0@	tp¯J MathType@MTEF@5@5@+=feaafiart1ev1aaatCvAUfKttLearuWrP9MDH5MBPbIqV92AaeXatLxBI9gBaebbnrfifHhDYfgasaacH8akY=wiFfYdH8Gipec8Eeeu0xXdbba9frFj0=OqFfea0dXdd9vqai=hGuQ8kuc9pgc9s8qqaq=dirpe0xb9q8qiLsFr0=vr0=vr0dc8meaabaqaciaacaGaaeqabaqabeGadaaakeaadaqdaaqaaiabdsha0jabdchaWbaadaWgaaWcbaGaemOsaOeabeaaaaa@30E0@	*J*_0_	tpJ0 MathType@MTEF@5@5@+=feaafiart1ev1aaatCvAUfKttLearuWrP9MDH5MBPbIqV92AaeXatLxBI9gBaebbnrfifHhDYfgasaacH8akY=wiFfYdH8Gipec8Eeeu0xXdbba9frFj0=OqFfea0dXdd9vqai=hGuQ8kuc9pgc9s8qqaq=dirpe0xb9q8qiLsFr0=vr0=vr0dc8meaabaqaciaacaGaaeqabaqabeGadaaakeaacqWG0baDcqWGWbaCdaWgaaWcbaGaemOsaO0aaSbaaWqaaiabicdaWaqabaaaleqaaaaa@31F5@	*N*_*t*_	Server	tp¯R MathType@MTEF@5@5@+=feaafiart1ev1aaatCvAUfKttLearuWrP9MDH5MBPbIqV92AaeXatLxBI9gBaebbnrfifHhDYfgasaacH8akY=wiFfYdH8Gipec8Eeeu0xXdbba9frFj0=OqFfea0dXdd9vqai=hGuQ8kuc9pgc9s8qqaq=dirpe0xb9q8qiLsFr0=vr0=vr0dc8meaabaqaciaacaGaaeqabaqabeGadaaakeaadaqdaaqaaiabdsha0jabdchaWbaadaWgaaWcbaGaemOuaifabeaaaaa@30F0@	tp¯J MathType@MTEF@5@5@+=feaafiart1ev1aaatCvAUfKttLearuWrP9MDH5MBPbIqV92AaeXatLxBI9gBaebbnrfifHhDYfgasaacH8akY=wiFfYdH8Gipec8Eeeu0xXdbba9frFj0=OqFfea0dXdd9vqai=hGuQ8kuc9pgc9s8qqaq=dirpe0xb9q8qiLsFr0=vr0=vr0dc8meaabaqaciaacaGaaeqabaqabeGadaaakeaadaqdaaqaaiabdsha0jabdchaWbaadaWgaaWcbaGaemOsaOeabeaaaaa@30E0@	*J*_0_	tpJ0 MathType@MTEF@5@5@+=feaafiart1ev1aaatCvAUfKttLearuWrP9MDH5MBPbIqV92AaeXatLxBI9gBaebbnrfifHhDYfgasaacH8akY=wiFfYdH8Gipec8Eeeu0xXdbba9frFj0=OqFfea0dXdd9vqai=hGuQ8kuc9pgc9s8qqaq=dirpe0xb9q8qiLsFr0=vr0=vr0dc8meaabaqaciaacaGaaeqabaqabeGadaaakeaacqWG0baDcqWGWbaCdaWgaaWcbaGaemOsaO0aaSbaaWqaaiabicdaWaqabaaaleqaaaaa@31F5@	*N*_*t*_
zhang-server [45]	-	71	19.5	68	85	ORFeus-2 [17]	61	62	45.6	61	83
Sam_t06_server [27]	-	69	18.9	67	85	phyre-1 [19]	-	62	41.2	58	77
hhpred2 [46]	68	68	49.5	59	85	*shub [11]*	65	62	51.4	58	83
fams [26]	-	68	21.6	67	85	foldpro [10]	-	62	25.8	60	85
*pmodeller6 [2]*	69	68	45.9	63	85	bilab-enable	26	62	27.5	60	84
famsd [26]	-	67	45.9	61	85	loopp [32]	55	62	33.1	60	85
circle [26]	-	67	37.4	63	85	protinfo [22]	-	62	83.5	50	85
raptoress [23]	-	67	30.6	63	85	fugue [25]	-	61	37.0	58	85
*pcons6 [2]*	69	67	46.9	63	84	phyre-2 [19]	55	60	37.1	60	83
metatasser [41]	-	67	29.8	64	85	3dpro [10]	-	59	34.6	58	85
hhpred1 [46]	64	67	54.1	59	85	karypis.srv.2 [39]	-	59	32.9	56	85
raptor [23]	37	67	37.9	62	85	fugmod [25]	55	58	24.9	57	79
robetta [37]	66	67	45.9	62	85	keasar-server [35]	-	58	74.5	53	81
sam-t02 [34]	-	66	25.0	62	82	sam-t99 [40]	-	58	11.9	58	61
karypis.srv [39]	-	66	31.4	59	83	nn_put_lab [47]	-	57	29.4	56	80
bayeshh [46]	64	65	34.5	63	85	rokky [42]	-	57	48.9	54	84
sp4 [31]	64	65	30.1	62	85	3D-PSSM [19]	52	57	44.0	56	84
uni-eid_bnmx	-	65	58.1	59	85	3d-jigsaw [24]	39	56	40.1	55	85
hhpred3 [46]	63	65	33.1	63	85	pdbblast [18]	56	56	54.1	55	81
BasD [12]	65	65	46.4	62	84	uni-eid_expm	54	55	41.0	52	67
sp3 [31]	64	65	45.9	61	85	3d-jigsaw_populus [24]	43	54	35.1	54	85
sparks2 [31]	62	65	35.4	63	85	3d-jigsaw_recom [24]	30	54	40.8	54	85
raptor-ace [23]	60	65	45.9	61	85	huber-torda-server [30]	34	54	23.4	53	82
uni-eid_sfst	32	65	40.2	59	83	forecast-s	-	53	39.5	53	84
mgen-3d [38]	-	64	29.9	63	83	distill [43]	32	44	31.4	44	85
*ffas03 [13]*	65	64	44.6	62	85	ma-opus-server2	-	44	36.9	41	55
function [26]	-	64	46.2	61	85	cphmodels [48]	-	40	43.2	40	41
ma-opus-server	-	64	43.4	60	85	frankenstein [33]	28	35	34.6	34	45
beautshot	48	63	47.8	61	85	gtg [28]	-	30	20.9	30	34
forte1 [36]	-	63	45.2	60	85	panther2	-	28	36.5	28	34
mgenthreader [38]	63	63	51.1	59	84	mig_frost [49]	-	23	43.4	23	34
forte2 [36]	-	63	48.2	60	85	abipro	-	16	20.2	14	84
nfold [16]	56	63	51.6	58	85	fugsa [25]	1	1	129.8	1	1
beautshotbase	48	63	41.8	61	83	mig_frost_flex [49]	1	1	73.0	1	2
caspita-fox	58	62	44.9	58	83	panther3	-	1	74.8	1	4
genesilicometaserver [33]	-	62	52.6	58	83	pomysl	-	1	0.0	0	50
protinfo-ab [22]	-	62	34.8	60	83	fpsolver-server [50]	-	0	0.0	0	81
*inub [44]*	63	62	51.2	59	85	karypis.srv.4 [39]	-	0	0.0	0	77

The *J*_0 _scores listed in Table 2 indicate the lowest 3D-Jury score seen before a bad model was encountered from the indicated server. In other words, no bad model above *J*_0 _score was seen in the test model set of the server. *J*_0 _scores are of practical value: they can be used as server-specific score thresholds, since a score above *J*_0 _is likely to indicate a good model.

### 3D-Jury scoring of user models

In order to encourage model selection and refinement using 3D-Jury, we introduced a new feature: instant 3D-Jury scoring of user models. This feature, available for any completed job by selecting the job in the Queue and uploading a model, enables the user to score a set of models and obtain a ranking based on the 3D-Jury score. Pop-up hints and an on-line tutorial [14], available from the job page, offer help with this new feature.

## Conclusion

In this report we present the evaluation of 3D-Jury [7] on models gathered in CASP7. We found good correlation between the 3D-Jury score and a model quality measure: the number of correctly predicted residues. This correlation can be used to predict important model features such as the number of correctly positioned residues. Using Figure 2, 3D-Jury scores can be translated to the estimated number of correctly predicted residues. We plan to upgrade the on-line 3D-Jury to provide the 90%, 95% and 99% prediction intervals for the number of correctly predicted residues automatically.

3D-Jury, in general, also appears to boost server predictions by identifying better models. Our results show that 3D-Jury performs best when all models of all servers are used to calculate the *J score*. This option, however, is not feasible in the Meta Server since many of the servers participating in CASP7 are not currently available on-line. Nevertheless, 3J_1,*A*_, the provided on-line default presents a reasonable choice. We found that 3D-Jury scores can be used as generic reliability scores, an especially important feature for models that are not provided with such values. We have also extracted serverwise 3D-Jury score thresholds to help identifying reliable models. We report the release of a new Meta Server feature: instant 3D-Jury scoring of uploaded user models.

3D-Jury remains to be a valuable tool in the hands of protein structure modellers. Its ability to pinpoint the best server models is founded by the results of our analysis.

## Methods

### Test model set

In order to assess 3D-Jury we downloaded the complete set of server structure predictions from the Protein Structure Prediction Center [15]. Predictions from our partner servers (BasD [12], ffas03 [13], inub [11], mgenthreader [16], ORFeus-2 [17], pdbblast [18] and 3D-PSSM [19]) were added if missing.

Servers that predicted less than two targets and/or returned only one model for each target were excluded from the server model ranking tests (reported in Table 1). The resulting set contains 25,215 models for 85 targets from 59 servers – a 5 models per server average.

Models with *Jscore *= 0 were excluded from all correlation and regression analyses.

Server reliability scores (*Rscore*) that anti-correlate with model quality were multiplied by -1.

### Model quality measures

MaxSub [8] score and NCα≤3.5Å
 MathType@MTEF@5@5@+=feaafiart1ev1aaatCvAUfKttLearuWrP9MDH5MBPbIqV92AaeXatLxBI9gBaebbnrfifHhDYfgasaacH8akY=wiFfYdH8Gipec8Eeeu0xXdbba9frFj0=OqFfea0dXdd9vqai=hGuQ8kuc9pgc9s8qqaq=dirpe0xb9q8qiLsFr0=vr0=vr0dc8meaabaqaciaacaGaaeqabaqabeGadaaakeaacqWGobGtdaWgaaWcbaGaem4qam0aaSbaaWqaaGGaciab=f7aHbqabaWccqGHKjYOcqaIZaWmcqGGUaGlcqaI1aqntCvAUfeBSjuyZL2yd9gzLbvyNv2CaeHbhv2BYDwAHbaceiGaa4xXaaqabaaaaa@413E@ (defined below) were used to measure the quality of models. Maxsub returns a score between 0.0 (incorrect prediction) and 1.0 (perfect prediction). In this study the score was multiplied by 10.0 as is customary on the 3D-Jury web pages [20]. We say that models with *MaxS *> 0 are good, while models with *MaxS *= 0 are bad.

NCα≤3.5Å
 MathType@MTEF@5@5@+=feaafiart1ev1aaatCvAUfKttLearuWrP9MDH5MBPbIqV92AaeXatLxBI9gBaebbnrfifHhDYfgasaacH8akY=wiFfYdH8Gipec8Eeeu0xXdbba9frFj0=OqFfea0dXdd9vqai=hGuQ8kuc9pgc9s8qqaq=dirpe0xb9q8qiLsFr0=vr0=vr0dc8meaabaqaciaacaGaaeqabaqabeGadaaakeaacqWGobGtdaWgaaWcbaGaem4qam0aaSbaaWqaaGGaciab=f7aHbqabaWccqGHKjYOcqaIZaWmcqGGUaGlcqaI1aqntCvAUfeBSjuyZL2yd9gzLbvyNv2CaeHbhv2BYDwAHbaceiGaa4xXaaqabaaaaa@413E@ is the number of C_*α *_atoms that are predicted within 3.5 Å from their respective locations in the solved structure, as reported by the MaxSub tool [8] operating on the C_*α *_atoms of the structures compared. We say that NCα≤3.5Å
 MathType@MTEF@5@5@+=feaafiart1ev1aaatCvAUfKttLearuWrP9MDH5MBPbIqV92AaeXatLxBI9gBaebbnrfifHhDYfgasaacH8akY=wiFfYdH8Gipec8Eeeu0xXdbba9frFj0=OqFfea0dXdd9vqai=hGuQ8kuc9pgc9s8qqaq=dirpe0xb9q8qiLsFr0=vr0=vr0dc8meaabaqaciaacaGaaeqabaqabeGadaaakeaacqWGobGtdaWgaaWcbaGaem4qam0aaSbaaWqaaGGaciab=f7aHbqabaWccqGHKjYOcqaIZaWmcqGGUaGlcqaI1aqntCvAUfeBSjuyZL2yd9gzLbvyNv2CaeHbhv2BYDwAHbaceiGaa4xXaaqabaaaaa@413E@ gives the number of correctly predicted residues.

### 3D-Jury model scoring

The 3D-Jury score of a model *M *is calculated by first comparing *M *to a set of other models available to the system for the same target. The way these other models are selected is a tunable parameter of 3D-Jury. *M *is compared to each selected model, and a pairwise similarity score (*S*_*M,i*_, for pair *i*) is assigned that equals to the number of respective C_*α *_atoms that are within 3.5 Å of each other after optimal superposition of the structures represented by their the C_*α *_atoms. MaxSub [8] is used to carry out this step. In case a pairwise similarity score falls below a certain cutoff value, it is set to zero. The 3D-Jury score (Jscore) of model *M *is the sum of its pairwise similarity scores divided by the number of these scores (*n*) + 1 [7]: JscoreM=∑inSM,in+1
 MathType@MTEF@5@5@+=feaafiart1ev1aaatCvAUfKttLearuWrP9MDH5MBPbIqV92AaeXatLxBI9gBaebbnrfifHhDYfgasaacH8akY=wiFfYdH8Gipec8Eeeu0xXdbba9frFj0=OqFfea0dXdd9vqai=hGuQ8kuc9pgc9s8qqaq=dirpe0xb9q8qiLsFr0=vr0=vr0dc8meaabaqaciaacaGaaeqabaqabeGadaaakeaacqWGkbGscqWGZbWCcqWGJbWycqWGVbWBcqWGYbGCcqWGLbqzdaWgaaWcbaGaemyta0eabeaakiabg2da9maalaaabaWaaabCaeaacqWGtbWudaWgaaWcbaGaemyta0KaeiilaWIaemyAaKgabeaaaeaacqWGPbqAaeaacqWGUbGBa0GaeyyeIuoaaOqaaiabd6gaUjabgUcaRiabigdaXaaaaaa@440E@.

#### 3D-Jury parameters

3D-Jury offers three tunable parameters: the list of servers to draw models from for pairwise score calculation; the method of server model selection (applicable in case of multiple available models, the name of the method is shown in italics): *first *model, most similar (in terms of *S*_*M,i*_) *one*, or *all *models; and the pairwise similarity score cutoff [7]. In this analysis we used the publicly available BasD [12], ffas03 [13], inub [11], mgenthreader [16], ORFeus-2 [17], pdbblast [18] and 3D-PSSM [19] as default servers and a constant similarity cutoff of 40 in order to simulate regular on-line use of the service.

### 3D-Jury operating modes

The four operating modes of 3D-Jury used in this report are: 3J_1,*A *_– uses *one *model of the *default *servers (a mode typical for on-line predictions); 3J_*a,A *_– *all *models of *default *servers; 3J_1,*C *_– *one *model of *all *servers; 3J_*a,C *_– *all *models of *all *servers.

### Measures for comparing model selection methods

#### *Q*_% _– 3D-Jury vs. original server

Q%=(∑MaxSj∑MaxSs−1)×100
 MathType@MTEF@5@5@+=feaafiart1ev1aaatCvAUfKttLearuWrP9MDH5MBPbIqV92AaeXatLxBI9gBaebbnrfifHhDYfgasaacH8akY=wiFfYdH8Gipec8Eeeu0xXdbba9frFj0=OqFfea0dXdd9vqai=hGuQ8kuc9pgc9s8qqaq=dirpe0xb9q8qiLsFr0=vr0=vr0dc8meaabaqaciaacaGaaeqabaqabeGadaaakeaacqWGrbqudaWgaaWcbaGaeiyjaucabeaakiabg2da9maabmaabaWaaSaaaeaadaaeabqaaiabd2eanjabdggaHjabdIha4jabdofatnaaBaaaleaacqWGQbGAaeqaaaqabeqaniabggHiLdaakeaadaaeabqaaiabd2eanjabdggaHjabdIha4jabdofatnaaBaaaleaacqWGZbWCaeqaaaqabeqaniabggHiLdaaaOGaeyOeI0IaeGymaedacaGLOaGaayzkaaGaey41aqRaeGymaeJaeGimaaJaeGimaadaaa@49B4@

∑*MaxS*_*j *_– sum of MaxSub scores of models selected by 3J_1,*A*_

∑*MaxS*_*s *_– sum of MaxSub scores of the server's first models

#### Q%max
 MathType@MTEF@5@5@+=feaafiart1ev1aaatCvAUfKttLearuWrP9MDH5MBPbIqV92AaeXatLxBI9gBaebbnrfifHhDYfgasaacH8akY=wiFfYdH8Gipec8Eeeu0xXdbba9frFj0=OqFfea0dXdd9vqai=hGuQ8kuc9pgc9s8qqaq=dirpe0xb9q8qiLsFr0=vr0=vr0dc8meaabaqaciaacaGaaeqabaqabeGadaaakeaacqWGrbqudaqhaaWcbaGaeiyjaucabaGaemyBa0MaemyyaeMaemiEaGhaaaaa@32FD@ – 'best model' vs. original server

Q%max=(∑max(MaxS)∑MaxSs−1)×100
 MathType@MTEF@5@5@+=feaafiart1ev1aaatCvAUfKttLearuWrP9MDH5MBPbIqV92AaeXatLxBI9gBaebbnrfifHhDYfgasaacH8akY=wiFfYdH8Gipec8Eeeu0xXdbba9frFj0=OqFfea0dXdd9vqai=hGuQ8kuc9pgc9s8qqaq=dirpe0xb9q8qiLsFr0=vr0=vr0dc8meaabaqaciaacaGaaeqabaqabeGadaaakeaacqWGrbqudaqhaaWcbaGaeiyjaucabaGaemyBa0MaemyyaeMaemiEaGhaaOGaeyypa0ZaaeWaaeaadaWcaaqaamaaqaeabaGaemyBa0MaemyyaeMaemiEaGhaleqabeqdcqGHris5aOWaaeWaaeaacqWGnbqtcqWGHbqycqWG4baEcqWGtbWuaiaawIcacaGLPaaaaeaadaaeabqaaiabd2eanjabdggaHjabdIha4jabdofatnaaBaaaleaacqWGZbWCaeqaaaqabeqaniabggHiLdaaaOGaeyOeI0IaeGymaedacaGLOaGaayzkaaGaey41aqRaeGymaeJaeGimaaJaeGimaadaaa@520E@

∑*max*(*MaxS*) – sum of the server's highest, best MaxSub scores per target

∑*MaxS*_*s *_– sum of MaxSub scores of the server's first models

### Receiver operating characteristic (ROC) analysis

We performed a ROC analysis adapted for CASP and Livebench [18] model evaluation for each server. Server models were ordered by the original reliability score (*Rscore*, when available), or the 3D-Jury score (*Jscore*). The highest scoring models for each target were collected into separate sets *M*_*R *_and *M*_*J*_, corresponding to the *Rscore *or *Jscore *used for ordering. Models in both sets were ordered by their respective scores. Good models (*MaxS *> 0) were labelled positive, bad models (*MaxS *= 0) were labelled negative. Using *Rscore *or *Jscore *as the discrimination threshold, we plotted the number of true positives (*tp*) versus the number of false positives (*fp*) on the [0 – 10] *fp *range. This was to take into account the absolute number of targets predicted by the servers, focusing on the hardest targets. We used the number of true positives averaged over the [0 – 10] false positive range as a quality measure for the reliability scores, the higher values indicating better reliability scores.

### Statistics and figures

Reported correlation coefficients are significant at the 95% significance level.

Statistics and figures were prepared using R [21].

## Availability and requirements

**Project name: **Meta Server/3D-Jury

**Project home page: **

**Operating system: **Linux

**Programming language: **Perl

**Other requirements: **SQL server, web server, mail server, procmail

**Licence: **the web service is freely accessible to everybody

## Authors' contributions

LK carried out the statistical analysis of the data, programmed the user model scoring feature and prepared the first draft of the manuscript. LR conceived of the study, coordinated it and revised this manuscript.
